# Ethylene Glycol Poisoning Without Fomepizole in a Regional Australian Intensive Care Unit: The Lactate Gap as the Diagnostic Key and the Consequences of Nasogastric Ethanol Therapy

**DOI:** 10.1002/ccr3.73698

**Published:** 2026-10-06

**Authors:** Jacob Athans, Prashant Maan

**Affiliations:** ^1^ Department of Intensive Care Medicine Hervey Bay Hospital Hervey Bay Queensland Australia; ^2^ University of Queensland Brisbane Queensland Australia

**Keywords:** acidosis, continuous renal replacement therapy, ethylene glycol, fomepizole, poisoning

## Abstract

A 62‐year‐old man presented comatose with pH 6.88 and no ingestion history. A point‐of‐care lactate above 31 mmol/L with a laboratory lactate of 0.8 mmol/L revealed a lactate gap, diagnosing ethylene glycol poisoning despite a near‐normal osmolar gap. High‐dose continuous renal replacement therapy corrected the acidosis; he survived.

## Key Clinical Message

In late‐presenting ethylene glycol poisoning the osmolar gap may be near‐normal; a lactate gap, a markedly elevated point‐of‐care lactate with a normal laboratory lactate, reveals the diagnosis. Where fomepizole is unavailable, high‐dose continuous renal replacement therapy is the key intervention, and nasogastric ethanol carries practical risks in ventilated patients.

## 1 Introduction

Ethylene glycol (EG) poisoning causes severe high‐anion gap metabolic acidosis (HAGMA) through sequential metabolism to glycolate and oxalate, the latter precipitating as calcium oxalate crystals causing acute kidney injury (AKI) and hypocalcemia [1, 2]. In late presentations, the osmolar gap may be near‐normal once EG is metabolized; the lactate gap, defined as the discordance between point‐of‐care (POC) and laboratory lactate caused by glycolate cross‐reactivity with lactate oxidase assays, is a more temporally reliable diagnostic marker [3, 4]. Fomepizole is the preferred alcohol dehydrogenase (ADH) inhibitor, though ethanol remains in use where fomepizole is unavailable [5, 6]. We report a case of severe late‐presentation EG poisoning managed without fomepizole in a regional Queensland ICU. This case is reported in accordance with the CARE guidelines.

## 2 Case History and Examination

### 2.1 Presentation and Differential Diagnosis

A 62‐year‐old man with a background of heavy alcohol use disorder was brought by ambulance to a regional Queensland hospital after being found unresponsive at home. No witnessed history was available. No antifreeze or toxic substance was identified at the scene, and the patient was obtunded and unable to provide history on arrival. He vomited and aspirated before intubation, with the aspiration occurring at the scene before advanced airway management was achieved.

The initial picture was striking: coma, extreme HAGMA, near‐normal blood pressure, AKI, markedly elevated POC lactate, and ionized hypocalcemia (iCa 0.90 mmol/L). This combination, and particularly the oxalate‐driven hypocalcemia characteristic of EG toxicity, raised EG early, but the differential remained broad. Septic and cardiogenic shock were considered and excluded on bedside echocardiography, which showed normal biventricular function with hyperdynamic, vasodilated hemodynamics. A more swollen left lower limb raised vascular or compartment syndrome, but distal pulses were present in all four limbs and compartment pressures were normal. Creatine kinase was 1600 U/L, insufficient to explain the acidosis by rhabdomyolysis. Salicylate and paracetamol were normal, and metformin toxicity was considered given the apparent lactic acidosis.

The decisive finding came when the laboratory serum lactate returned at 19:00. The POC arterial blood gas lactate was markedly elevated (initially 28.0 mmol/L, subsequently > 31.0 mmol/L, the upper reporting limit of the analyzer), yet the contemporaneous laboratory serum lactate was 0.8 mmol/L. This lactate gap of greater than 30 mmol/L was recognized as consistent with glycolate cross‐reactivity with the lactate oxidase assay, elevating toxic alcohol to the primary diagnosis.

### 2.2 Admission Biochemistry

Initial POC blood gas at the peripheral hospital (14:26) demonstrated: pH 6.88, *p*CO_2_ 64 mmHg, *p*O_2_ 75 mmHg (room air), bicarbonate 12 mmol/L, base excess −22.5 mmol/L, POC lactate 28.0 mmol/L, anion gap 28 mEq/L, ionized calcium 1.03 mmol/L, and creatinine 121 μmol/L. Following intubation and transfer, the ICU admission gas on FiO_2_ 0.60 showed pH 7.07, *p*CO_2_ 41 mmHg, *p*O_2_ 80 mmHg, anion gap 31 mEq/L, ionized calcium 0.90 mmol/L, and POC lactate > 31.0 mmol/L. Formal laboratory investigations drawn at 18:30 demonstrated a serum lactate of 0.8 mmol/L (returned at 19:00), creatinine 214 μmol/L, total calcium 1.68 mmol/L (albumin‐corrected 2.16 mmol/L), phosphate 3.23 mmol/L, CK 2310 U/L, fibrinogen 8.7 g/L, white cell count 29.4 × 10^9^/L, CRP 309 mg/L, GGT 393 U/L, and serum ethanol < 3 mmol/L. Measured osmolality was 344 mOsm/kg; with a laboratory‐calculated osmolality of 339 mOsm/kg, the osmolar gap was 5 mOsm/kg, essentially normal, consistent with late presentation in which the parent compound had largely been metabolized (Table 1). ROTEM demonstrated a hypercoagulable profile with FIBTEM A10 27 mm and EXTEM A10 75 mm, concordant with the elevated fibrinogen.

**TABLE 1:** Serial biochemical values from peripheral hospital presentation to early ICU admission.

Parameter	Peripheral hospital (14:26)	ICU 18:30 labs
pH	6.88	7.07
*p*CO_2_ (mmHg)	64	41
*p*O_2_ (mmHg)	75	80
Bicarbonate (mmol/L)	12	13
Base excess (mmol/L)	−22.5	−17.8
Anion gap (mEq/L)	28	31
POC lactate (mmol/L)	28.0	> 31.0
Serum lactate (mmol/L)	Not taken	0.8
Lactate gap (mmol/L)	Not taken	> 30
Ionized Ca (mmol/L)	1.03	0.90
Total Ca (mmol/L)	2.38	1.68
Creatinine (μmol/L)	121	214
Sodium (mmol/L)	146	153
Osmolality measured (mOsm/kg)	Not taken	344
Osmolality calculated (mOsm/kg)	332	339
Osmolar gap (mOsm/kg)	NA	5
FiO_2_	0.21 (room air)	0.60

*Note: p*O_2_ at the peripheral hospital was on room air; the ICU value was on FiO_2_ 0.60. The serum lactate of 0.8 mmol/L returned at 19:00 and, against the contemporaneous point‐of‐care lactate above 31.0 mmol/L from the 18:30 gas, revealed the lactate gap. Osmolar gap calculated as measured minus laboratory‐calculated osmolality.

Abbreviation: POC, point of care.

The initial volatile screen specimen was collected at 13:45 as part of the presentation workup. EG was added to this stored specimen retrospectively, once the lactate gap had raised toxic alcohol, and was processed by the state reference laboratory, returning 58 h after collection at 5.8 mmol/L. This retrospective result played no role in acute management, which proceeded on clinical and surrogate biochemical grounds. Once the lactate gap was recognized, EG was requested prospectively to monitor elimination, and the levels are shown in Table 2. The patient subsequently denied ingesting antifreeze and remained delirious for many days during the ward admission; a definitive ingestion history was never established.

**TABLE 2:** Ethylene glycol and ethanol levels over the treatment course.

Date/time	Ethylene glycol (mmol/L)	Ethanol (mmol/L)	Comment
Mar 1, 13:45 (collected)	5.8	< 1.0	Presentation specimen; EG assay added retrospectively after lactate gap; returned 58 h later
Mar 1, 23:30	2.5	< 1.0	On CVVHDF (commenced 19:30); prospective monitoring
Mar 2, 09:30	< 1.0	2.8	EG undetectable ~14 h post‐CVVHDF; ethanol weaning
Mar 2 (later)	< 1.0	26.6	Therapeutic ethanol confirmed

*Note:* The 13:45 specimen was analyzed for ethylene glycol retrospectively; subsequent levels were requested prospectively once the lactate gap was recognized. Results were returned from the state reference laboratory with delays of up to 58 h and were not available in real time to guide acute management.

## 3 Investigations and Management

### 3.1 Toxicology Consultation and Fomepizole Availability

The toxicology service was consulted. Before the lactate gap was recognized it did not favor presumptive treatment, noting that confirmed toxic alcohol poisoning was uncommon locally. Once the lactate gap identified toxic alcohol, toxicology concurred with commencing ethanol. Fomepizole was not held on site and could not be accessed within the required timeframe, so ethanol, prepared in advance by pharmacy, was the practical choice.

### 3.2 Management

CVVHDF was commenced at 19:30, 1 h after the ICU admission gas, on two indications: severe metabolic acidosis and suspected toxic alcohol poisoning following identification of the lactate gap. It was delivered via a Prismaflex ST150 set at an effluent dose of 40 mL/kg/h, substantially above the standard 25–30 mL/kg/h prescription, to maximize glycolate, oxalate, and residual EG clearance (Table 3). Because 800 mL/h. Unfractionated heparin was selected as the anticoagulation modality. Citrate anticoagulation was explicitly avoided: in EG poisoning, hypocalcemia is driven by ongoing calcium oxalate precipitation, and administering citrate, which further chelates ionized calcium in the extracorporeal circuit, in the context of an already severely depleted ionized calcium of 0.90 mmol/L would risk compounding systemic hypocalcemia [7]. Aggressive intravenous calcium chloride replacement was required throughout. Filter clotting was encountered and required heparin escalation, consistent with the hypercoagulable milieu reflected by hyperfibrinogenemia and the ROTEM profile. CVVHDF was continued for 72 h in total; the initial indication was toxin clearance and correction of acidosis, and treatment was continued for renal support as the patient became oliguric with established AKI.

**TABLE 3:** Extracorporeal treatment (CVVHDF) parameters.

Parameter	Value
Modality	CVVHDF
Filter	ST150 (AN69, 1.5 m^2^)
Blood flow	200 mL/min
Dialysate flow	1800 mL/h
Replacement fluid	Pre‐dilution 800 mL/h; post‐dilution 200 mL/h
Net ultrafiltration	Nil
Total effluent	2800 mL/h
Delivered dose	40 mL/kg/h (70 kg)
Anticoagulation	Unfractionated heparin
Duration	72 h (initial toxin clearance and acidosis correction; continued for renal support during oliguric AKI)

*Note:* Ethylene glycol fell from 5.8 mmol/L (retrospective pretreatment baseline) to 2.5 mmol/L at 4 h and undetectable by about 14 h, an apparent on‐treatment half‐life of roughly 4–6 h. This is approximate: ethanol blockade and residual metabolism confound a pure dialytic clearance, and no paired circuit samples were obtained.

Abbreviation: CVVHDF, continuous veno‐venous hemodiafiltration.

Nasogastric ethanol 37% solution was commenced as ADH blockade at 125 mL/h. A therapeutic serum ethanol of 26.6 mmol/L was confirmed on Day 2, within the target range of 20–30 mmol/L [5]. However, after 24 h of administration, the patient developed progressive abdominal distension attributable to the cumulative volume of nasogastric ethanol (approximately 3 L over 24 h), compounded by preexisting aspiration injury and ileus. The abdominal distension may have contributed to worsening respiratory mechanics, increased ventilatory pressures, and hypoxemia in an already compromised respiratory system. By this point, the metabolic acidosis had substantially resolved on CVVHDF and the anion gap was normalizing, indicating clearance of glycolate and oxalate. Ethanol was therefore ceased, balancing the diminishing benefit of ongoing ADH blockade against the ongoing respiratory risk. EG had become undetectable on volatile drug screen by 09:30 on March 2, approximately 14 h after CVVHDF commencement, confirming successful elimination before ethanol cessation.

## 4 Outcome and Follow‐Up

The patient required 5 days of mechanical ventilation. Prolonged sedation and weaning were complicated by alcohol withdrawal delirium, requiring dexmedetomidine and benzodiazepine therapy. He was extubated on Day 5, discharged from the ICU on Day 7, and discharged from hospital on Day 21, 2 weeks after ICU discharge. The clinical course is summarized in Figure 1. Renal function improved progressively over the admission; creatinine at hospital discharge was 90 μmol/L, representing substantial recovery from the peak of 390 μmol/L during the ICU admission.

**FIGURE 1:**
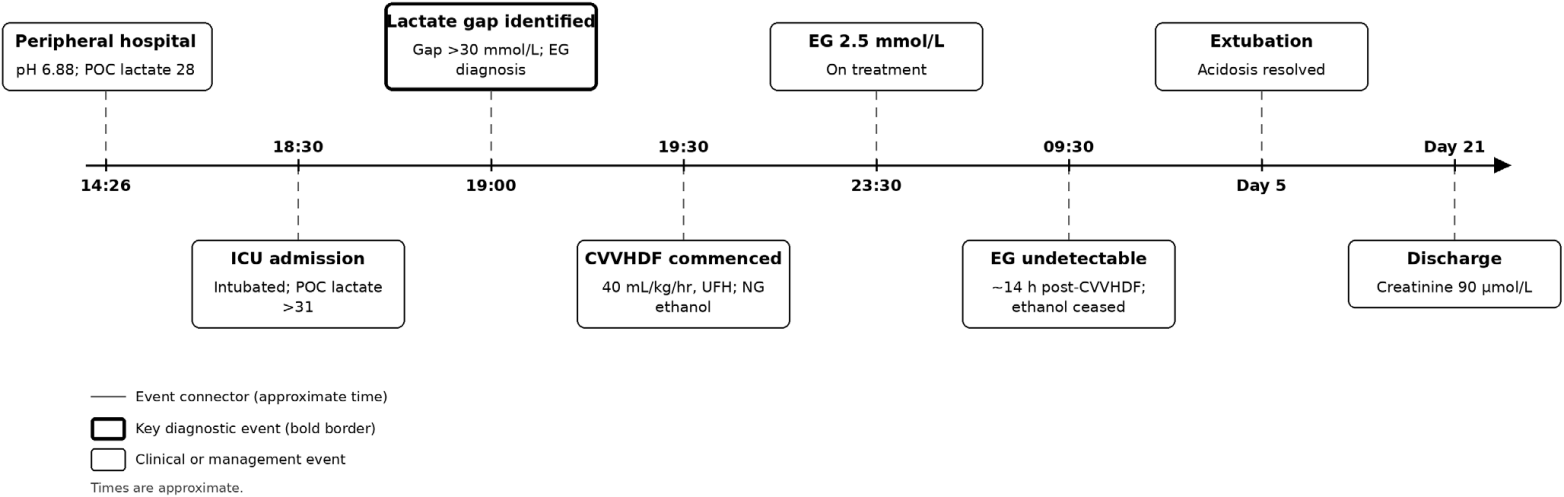
Clinical timeline from patient discovery to hospital discharge. Bold border denotes the pivotal diagnostic step. Times are approximate. CVVHDF, continuous veno‐venous hemodiafiltration; EG, ethylene glycol; ICU, intensive care unit; NG, nasogastric; POC, point of care; UFH, unfractionated heparin.

## 5 Discussion

This case combines a delayed presentation with a near‐normal osmolar gap, in which diagnosis rested almost entirely on an extreme lactate gap, management in a regional Australian ICU without fomepizole, and survival from a pH of 6.88. It raises four practical issues: the approach to a broad differential, the lactate gap in late EG presentation, fomepizole as an alternative to ethanol, and the competing priorities created by ethanol therapy in a ventilated patient with aspiration injury.

### 5.1 Diagnostic Reasoning in Undifferentiated Severe Acidosis

The normal osmolar gap of 5 mOsm/kg at ICU admission would typically lower suspicion for toxic alcohol under conventional teaching. However, a normal osmolar gap does not exclude EG poisoning in a delayed presentation: as EG is metabolized to organic acids, the parent compound that generates the osmolar gap is consumed, and the anion gap rises in its place [8]. The anion gap of 28–31 mEq/L with a near‐normal osmolar gap here is consistent with an intermediate‐to‐late presentation, a pattern that is well described but under‐appreciated, particularly at peripheral hospitals.

The systematic exclusion of alternative diagnoses, normal biventricular function on echocardiography, normal distal vascular assessment, normal salicylate and paracetamol levels, and CK insufficient for the degree of acidosis progressively narrowed the differential and elevated toxic alcohol. The lactate gap was then the decisive finding. A POC ABG lactate exceeding the analyzer upper limit (> 31.0 mmol/L) with a serum lactate of 0.8 mmol/L represents a gap of greater than 30 mmol/L [3, 4, 9, 10].

The discordance reflects assay chemistry, not a true lactatemia. POC and blood gas analyzers measure lactate with a lactate oxidase electrode, which also oxidizes glycolate and glyoxylate and therefore overreads. The laboratory value was normal because the central platform used a method specific for l‐lactate that is not meaningfully cross‐reactive with glycolate, and this interference is analyzer‐dependent [9]. The magnitude of the lactate gap correlates with the glycolate concentration but is not equal to it, and the relationship is not consistent or predictable across analyzers [3]; the gap is therefore a qualitative clue that should prompt confirmatory testing, not a quantitative measure of glycolate.

### 5.2 Fomepizole as an Alternative Agent

This case illustrates, at the individual patient level, the practical considerations that arise when ethanol rather than fomepizole is used as the ADH inhibitor. In a recent NSW cohort, fomepizole could have been advantageous in 18 of 28 ethanol‐treated patients (64%), including avoiding ICU admission (*n* = 8) and simplifying transport monitoring (*n* = 11) [6]. In this case, the use of ethanol rather than fomepizole had direct consequences. The hazard was not aspiration, since the endotracheal tube protected the airway and no further aspiration occurred, but the large enteral volume of 37% ethanol required to maintain therapeutic concentrations, which produced gastric and abdominal distension that worsened the hypoxia already established by the earlier at‐scene aspiration.

Fomepizole is superior to ethanol as an ADH inhibitor, and this case illustrates why it would have been preferable. It binds the enzyme far more avidly, giving more complete and reliable blockade; it has predictable pharmacokinetics with fixed 12‐hourly dosing and needs no serum‐level monitoring, whereas ethanol requires repeated levels to hold a target concentration and, here, a large and ultimately harmful enteral volume; and it causes neither sedation nor respiratory depression nor the emergence and withdrawal delirium that prolonged this patient's ventilation and weaning. It also has a demonstrated cost–benefit through easier monitoring, fewer complications, and reduced or avoided ICU admission and length of stay [5, 6].

The initial toxicology discussion weighed the local rarity of confirmed EG poisoning against the compelling biochemistry, illustrating the difficulty of empirical decisions when both ingestion history and confirmatory testing are unavailable. EG is present in many industrial and household products beyond antifreeze, so the absence of a history should not reduce suspicion when the biochemical pattern fits. With the confirmatory assay returning only after 58 h, regional clinicians must be prepared to diagnose and manage this condition without laboratory confirmation.

Fomepizole was not available anywhere in our region, and this reflects a wider gap for regional and rural Australian centers. Given its clinical advantages and demonstrated cost–benefit, health services and toxicology networks may wish to consider improved stocking and rapid‐access or distribution pathways, so that the antidote can reach patients in regional units when acute need arises. On both patient‐safety and resource grounds, such consideration appears warranted.

### 5.3 Ethanol Therapy: Risk–Benefit in a Ventilated Patient

The risk–benefit balance of nasogastric ethanol in this ventilated patient deserves emphasis. ADH blockade prevents ongoing metabolite generation from residual EG, but the osmolar gap was already near‐normal at presentation, so much of the EG had been metabolized before treatment and the incremental benefit at this late stage was uncertain. Against that limited benefit, the enteral volume required to maintain therapeutic concentrations (125 mL/h of 37% vodka, about 3 L over 24 h) produced progressive gastric and abdominal distension and deteriorating respiratory mechanics on a gastrointestinal tract already compromised by ileus.

The resolution of metabolic acidosis and normalization of the anion gap occurred on CVVHDF, not as a consequence of ethanol therapy. Extracorporeal treatment (ECTR) removes glycolate directly, the principal driver of acidosis, as well as residual EG and oxalate, all of which are small molecules with high sieving coefficients and efficient removal at 40 mL/kg/h effluent dose [2]. The ethanol was ceased at 24 h on clinical grounds, by which point CVVHDF had achieved the metabolic objectives. This sequence raises a legitimate question: in a late presentation with near‐normal osmolar gap and severe aspiration injury, is the risk–benefit balance of ethanol therapy acceptable when high‐dose CVVHDF is already running and achieving metabolite clearance? Fomepizole, with its predictable pharmacokinetics, ease of titration, and avoidance of large enteral volumes, may have simplified management by avoiding nasogastric ethanol.

### 5.4 ECTR: Indication, Modality and Rationale

ECTR was clearly indicated. The EXTRIP workgroup recommends ECTR in EG poisoning when coma, AKI, or an anion gap above 27 mmol/L is present, all of which applied here [11]. EXTRIP identifies intermittent hemodialysis as the preferred modality because its higher blood and dialysate flows achieve more rapid clearance and recommends continuous therapy as the alternative where intermittent hemodialysis is not available [11]. In our regional unit, intermittent hemodialysis was not available for a ventilated patient, and high‐dose CVVHDF was therefore used as the guideline‐sanctioned alternative, achieving metabolite clearance and correction of acidosis. This should not be read as CVVHDF being superior to hemodialysis; hemodialysis provides more rapid removal, and the continuous modality was selected here on grounds of availability rather than preference.

EG and its metabolites are well suited to extracorporeal removal. EG has a low molecular weight of approximately 62 Da, as do glycolate (approximately 76 Da) and oxalate (approximately 90 Da); the volume of distribution of EG is small, approximately 0.5–0.8 L/kg, close to total body water; and protein binding of all three is negligible. These properties, low molecular weight, small volume of distribution, and minimal protein binding, are what make EG and glycolate efficiently dialyzable, and both were formally assessed as dialyzable by EXTRIP [11]. Reporting of ECTR is strengthened by describing these determinants together with the circuit parameters (Table 3), in keeping with the EXTRIP reporting checklist [12]. Systemic EG fell from a retrospective pretreatment baseline of 5.8 to 2.5 mmol/L 4 h into CVVHDF and to undetectable by approximately 14 h, consistent with effective extracorporeal removal augmented by ADH blockade of further metabolism. A formal dialytic clearance could not be calculated, as paired circuit inlet and outlet samples were not obtained and concurrent ethanol blockade confounds interpretation; the apparent on‐treatment elimination half‐life was of the order of 4–6 h.

### 5.5 CRRT Anticoagulation: Individualized Decision‐Making

Regional citrate anticoagulation is recommended as the preferred modality for CRRT by KDIGO guidelines in the absence of contraindications [7, 13]. In this patient, systemic unfractionated heparin was used instead, as clinicians were concerned that citrate, which further chelates ionized calcium within the extracorporeal circuit, would increase an already substantial calcium replacement requirement in a patient with severe oxalate‐mediated hypocalcemia, with ionized calcium not sustained above 0.90 mmol/L despite ongoing supplementation. Whether citrate can be safely used in this setting, with closer monitoring and more aggressive calcium replacement, remains uncertain; anticoagulation strategy in such patients should be individualized according to clinical context and local expertise.

The hypercoagulable state, reflected by hyperfibrinogenemia of 8.7 g/L, elevated FIBTEM amplitudes on ROTEM, and the acute phase inflammatory response, explains the filter clotting encountered despite systemic heparin anticoagulation, and supports the use of viscoelastic testing to guide anticoagulation.

### 5.6 Implications for Practice

Three practical implications emerge from this case. First, a near‐normal or normal osmolar gap does not exclude EG poisoning in the context of severe unexplained HAGMA; late presentation with metabolic shift from osmolar to anion gap predominance must be anticipated. Second, the lactate gap, readily identifiable by comparing POC ABG lactate with simultaneous serum lactate, is a valuable diagnostic tool in regional settings where EG assays are unavailable in real time. Third, fomepizole represents an alternative to ethanol for ADH blockade and may avoid some of the practical difficulties of ethanol administration encountered in this case, particularly in ventilated patients. Local familiarity with fomepizole as a treatment option, and discussion of access pathways with toxicology services, may be of value in similar future presentations.

## 6 Conclusion

Survival from EG poisoning with arterial pH 6.88 is achievable in a regional Australian ICU when management is guided by systematic clinical reasoning in the absence of rapid confirmatory testing. The lactate gap was the decisive diagnostic finding, identified after progressive exclusion of alternative diagnoses. High‐dose CVVHDF was the primary therapeutic intervention that achieved metabolite clearance and acid–base restoration. Nasogastric ethanol, while providing ADH blockade, may have contributed to abdominal distension and respiratory compromise in a patient with concurrent aspiration injury. This case highlights fomepizole as an alternative agent that may avoid some of these practical difficulties and supports consideration of its role, and of improved regional access to it, in similar future presentations in regional Australian hospitals.

## Author Contributions

**Jacob Athans:** conceptualization, writing – original draft, writing – review and editing, resources. **Prashant Maan:** conceptualization, writing – original draft, visualization, writing – review and editing, supervision, resources.

## Funding

The authors have nothing to report.

## Ethics Statement

This report was assessed as non‐research activity and confirmed exempt from formal human research ethics review by the Queensland Health Coronial and Public Health Sciences Human Ethics Committee (EC00305; reference EX/2026/CPHS/128930).

## Consent

Written informed consent for publication of this case report and any accompanying images was obtained from the patient.

## Conflicts of Interest

The authors declare no conflicts of interest.

## Data Availability

The data that support the findings of this case report are not publicly available due to patient privacy restrictions but are available from the corresponding author on reasonable request.
